# Understanding Patients’ Intention to Use Digital Health Apps That Support Postdischarge Symptom Monitoring by Providers Among Patients With Acute Coronary Syndrome: Survey Study

**DOI:** 10.2196/34452

**Published:** 2022-03-07

**Authors:** Jinying Chen, Jessica G Wijesundara, Gabrielle E Enyim, Lisa M Lombardini, Ben S Gerber, Thomas K Houston, Rajani S Sadasivam

**Affiliations:** 1 Department of Population and Quantitative Health Sciences University of Massachusetts Chan Medical School Worcester, MA United States; 2 Department of Internal Medicine Wake Forest School of Medicine Winston-Salem, NC United States

**Keywords:** coronary, monitor, elder, health app, symptom, eHealth, mobile health, intention, barrier, facilitator

## Abstract

**Background:**

After hospital discharge, patients with acute coronary syndrome (ACS) often experience symptoms that prompt them to seek acute medical attention. Early evaluation of postdischarge symptoms by health care providers may reduce unnecessary acute care utilization. However, hospital-initiated follow-up encounters are insufficient for timely detection and assessment of symptoms. While digital health tools can help address this issue, little is known about the intention to use such tools in ACS patients.

**Objective:**

This study aimed to assess ACS patients’ intention to use digital health apps that support postdischarge symptom monitoring by health care providers and identify patient-perceived facilitators and barriers to app use.

**Methods:**

Using email invitations or phone calls, we recruited ACS patients discharged from a central Massachusetts health care system between December 2020 and April 2021, to participate in the study. Surveys were delivered online or via phone to individual participants. Demographics and access to technology were assessed. The intention to use a symptom monitoring app was assessed using 5-point Likert-type (from strongly agree to strongly disagree) items, such as “If this app were available to me, I would use it.” Responses were compared across demographic subgroups and survey delivery methods. Two open-ended questions assessed perceived facilitators and barriers to app use, with responses analyzed using qualitative content analysis.

**Results:**

Among 100 respondents (response rate 8.1%), 45 (45%) completed the survey by phone. The respondents were on average 68 years old (SD 13 years), with 90% (90/100) White, 39% (39/100) women, and 88% (88/100) having access to the internet or a mobile phone. Most participants (65/100, 65%) agreed or strongly agreed that they would use the app, among which 53 (82%) would use the app as often as possible. The percentage of participants with the intention to use the app was 75% among those aged 65-74 years and dropped to 44% among those older than 75 years. The intention to use was higher in online survey respondents (vs phone survey respondents; odds ratio 3.07, 95% CI 1.20-7.88) after adjusting for age and access to technology. The analysis of open-ended questions identified the following 4 main facilitators (motivations): (1) easily reaching providers, (2) accessing or providing information, (3) quickly reaching providers, and (4) consulting providers for symptoms, and the following 4 main barriers: (1) privacy/security concerns, (2) uncomfortable using technology, (3) user-unfriendly app interface, and (4) preference for in-person/phone care.

**Conclusions:**

There was a strong intention to use a symptom monitoring app postdischarge among ACS patients. However, this intent decreased in patients older than 75 years. The survey identified barriers related to technology use, privacy/security, and the care delivery mode. Further research is warranted to determine if such intent translates into app use, and better symptom management and health care quality.

## Introduction

The transition from inpatient care to home is challenging for patients with acute coronary syndrome (ACS) [1-3]. After hospital discharge, ACS patients often experience symptoms that prompt them to seek acute medical attention [2-6]. A large portion of these symptoms are noncardiac [3-7], and could be assessed and managed through close follow-up care in the outpatient setting to reduce unnecessary acute care utilization [3,5-7]. Symptom assessment and management are integral to transitional care [8-13], and are also part of the transitional care management services supported by Medicare [14]. However, hospital-initiated follow-up activities alone may be inadequate to detect symptoms in a timely fashion, as new or worsening symptoms may occur between the initial contact and the follow-up appointment [15]. Intensive transitional care programs offering multiple follow-up phone calls or home visits may better capture patient’s symptom episodes [11,12], but providing such thorough contact increases the need for staff resources and time, and can be challenging to scale up.

Digital health tools for symptom monitoring can support timely detection and evaluation of patients’ symptoms [16-20], and have been successfully integrated with routine cancer care [16,17,21-23]. Some tools allowed patients to report symptoms frequently or at any time [16,17]. However, in general, evidence about the feasibility and efficacy of using these tools to improve patient outcomes is still limited, especially in patients with ACS. A recent study analyzed data related to using a digital symptom monitoring tool (which allowed patients to self-rate and track their symptoms of fatigue) to enhance a patient-centered care intervention for cardiac rehabilitation [24]. This study found that the enhanced intervention improved patient-reported self-efficacy at 6 months postdischarge, compared with usual care (*P*=.01). However, only 39% of the patients in the intervention group chose to use the digital health tool.

More research is needed to understand the intention, barriers, and facilitators to digital health symptom monitoring in ACS patients. This is particularly true among older adults (≥65 years old) representative of the ACS population. Older adults have unique barriers in using technology, such as lack of knowledge and confidence, age-related changes or disabilities, and skepticism about the benefits [25,26]. Prior studies showed that most patients, including older adults, are ready to accept digital health tools for monitoring mental health conditions and symptoms, but the intention to use decreased with age [27,28]. Understanding these issues may help improve design, development, and adherence to digital symptom monitoring in ACS patients.

This study aimed to assess ACS patients’ intention to use digital health tools that support symptom monitoring by providers after hospital discharge. We conducted a survey, using both close-ended and open-ended questions, to assess the intention to use, the difference in the intention by patient characteristics (eg, age), and the facilitators and barriers of using these tools in this patient population. We also compared the intention to use between 2 survey delivery modes (online vs phone).

## Methods

### Study Design

We analyzed data collected through a survey using both close-ended and open-ended questions. The survey was delivered using 1 of the 2 modes (online surveys and phone calls) to ensure a balanced sample of participants who are comfortable or are not comfortable with the use of technology (ie, filling online surveys).

### Ethics Approval

The study was approved by the Institutional Review Board at the University of Massachusetts Chan Medical School. The ethics approval number (ie, the Institutional Review Board Docket Number) for this study is H00018298. The Institutional Review Board approved the use of informed verbal consent procedures. We obtained verbal informed consent from each participant by email or phone.

### Survey

The survey design was informed by prior literature on assessing participants’ intention to use digital interventions [29,30]. One researcher (with expertise in health informatics and implementation science) created the initial survey by adapting a subset of validated questions from a survey assessing participants’ intention to use mobile apps for COVID-19 symptom monitoring [30]. A cardiologist and 2 research team members (with training in public health and clinical research, respectively) reviewed the survey content and provided feedback on clarifying and simplifying the language of the introduction paragraph, the survey questions, and the response options.

The final survey (Multimedia Appendix 1) included 5 items to assess participants’ demographics (age, sex, and race) and access to technology (internet and smartphone), and 5 items (3 close-ended and 2 open-ended questions) related to the intention to use a hypothetical symptom monitoring app. The demographics questions and the open-ended questions were optional. Intention to use the app was assessed using a 5-point Likert-type (from “strongly agree” to “strongly disagree”) item (also called the intention-to-use question) as follows: “If this app were available to me, I would use it.” Participants who responded “strongly agree,” “agree,” or “neutral” to this item were prompted to respond to 2 additional items. The first item was a 5-point Likert-type item as follows: “I plan to use this app as often as necessary,” with response options ranging from “strongly agree” to “strongly disagree.” The second item was multiple-choice as follows: “I’d like the app to be designed as …,” with the following 3 options: “mobile app,” “web app,” and “other.” The 2 remaining open-ended questions collected free-text comments on the facilitators (ie, motivations) and barriers to using the app.

### Recruitment and Data Collection

We recruited patients from UMass Memorial Health Care, the largest health care system in Central Massachusetts, serving most patients hospitalized with cardiovascular diseases in this region.

Using information from electronic health records (EHRs), we identified adult patients (>18 years old) who were hospitalized for ACS (ICD-10 codes: I24.9, I21, I21.x, I21.xx, and I25.110) between January 2019 and December 2020, as eligible participants. Study data were collected and managed using REDCap electronic data capture tools hosted at the study institution [31,32].

We recruited participants with a 2-stage procedure, using emails and phone calls, respectively. In the first stage (December 2020), we emailed invitations to 782 candidate participants. Once a participant replied to the email to indicate their interest, we sent the online survey via a secure REDCap link to their email address. An unanswered survey was automatically disabled in REDCap 30 days after being sent to the participant. Recruitment stopped after more than 40 participants responded to the online survey.

In the second stage (January 2021 to April 2021), we recruited participants who did not have an email address listed in the EHR via phone calls. Recruitment calls were made to 448 candidate participants until the total number of responses to the survey (from both email and phone recruitment) met the target (N=100). For phone recruitment, we documented the reasons for declining participation. Participants recruited by phone were given the option to complete the survey online (using the same procedure described for stage 1) or via phone. For surveys answered by phone, a research staff member documented participants’ verbal responses in REDCap. Each survey participant (for both stages of participant recruitment) was provided a US $10 gift card to compensate for their time.

### Research Questions

The following 4 research questions were considered: (1) Do patients have the intention to use the app for symptom monitoring by providers? (Q1); (2) Is there a difference in the intention to use the app for symptom monitoring across subgroups characterized by participants’ characteristics, including age and access to technology? (Q2); (3) Is there a difference in the intention to use the app for symptom monitoring between participants responding to the survey online and those responding by phone? (Q3); and (4) What are the main factors that motivate or discourage patients’ use of an app for symptom monitoring by providers? (Q4).

### Statistical Analyses

Statistical analyses were performed using STATA/IC 15.1 (StataCorp). We first calculated descriptive statistics of participants’ characteristics and examined their distributions over the 2 survey delivery modes. We then analyzed the data to answer research questions 1 to 3. We used participants’ age information from the EHR, which has greater granularity than the survey responses, for these analyses.

First, we calculated descriptive statistics of participants’ responses to the 3 close-ended survey questions related to the intention to use the symptom monitoring app (Q1). Second, we examined the distribution of the intention to use over participants’ characteristics and access to technology (Q2). Third, we assessed the associations between survey delivery mode and participants’ intention to use the app (Q3), using multivariable logistic regression to adjust for potential confounding factors related to participants’ characteristics and access to technology. We identified the confounders based on the literature and the examination of the distribution of participants’ characteristics over survey delivery mode (*P*<.05). In addition, we combined access to the internet and access to a smartphone into 1 variable, access to technology, when adjusting for the association analysis because the 2 variables are interdependent (Fisher exact test *P*<.001).

When conducting analyses related to questions 2 and 3, we grouped the 5 response options of the intention-to-use question into 2 categories, with 1 representing “agree” and “strongly agree” and 0 representing the other options. In addition, we assigned numeric values to the 5 response options (1: strongly disagree, 2: disagree, 3: neutral, 4: agree, 5: strongly agree) and presented the summary statistics of the responses.

### Qualitative Analyses

To answer research question 4, we analyzed survey responses to the 2 open-ended survey questions through an iterative process using qualitative content analysis. Qualitative content analysis is a research method widely used to analyze written, verbal, or visual communication messages through the systematic coding and identification of themes or patterns [33-35]. Following established techniques [34,35], we carried on the analysis over 3 phases (ie, preparation, organizing, and reporting).

In the preparation phase, GEE (premed student with training in biology, neuroscience, and clinical research) read through the survey responses and assigned initial codes to the responses. JC (with expertise in health informatics and implementation science), JGW (with training in public health and health education), and GEE discussed the initial coding results and created the initial codebook. Using the initial codebook, GEE, JGW, and LML (with training in clinical research and neuroscience) coded all survey responses independently. Codes were assigned to each response (primarily single sentences), and double coding was allowed. The coded responses were discussed among GEE, JGW, LML, and JC to resolve discrepancies, and new codes were added when necessary. This process resulted in the final codebook (Multimedia Appendix 2), with 9 codes (4 categories) for the facilitator question and 8 codes (4 categories) for the barrier question. Based on the coding results, JC segmented survey responses into units that entail a single code. Most segments were single sentences; some were phrases or contained multiple sentences.

In the organizing phase, JC and JGW independently coded the segments using the final codebook. The intercoder agreement was 86% for the facilitator question and 87% for the barrier question. Discrepancies were discussed and resolved between JC and JGW to generate the final coding results.

In the reporting phase, we reported the definitions, frequencies, and representative quotes of codes and summarized key findings [34,35]. We identified the major barriers and facilitators to app use by considering code/category frequency and existing literature on health app use among patients or older adults, and through discussion in the research team. In addition, we compared the most salient facilitators and barriers for the following 2 age groups: younger and older than 65 years of age.

## Results

### Participant Characteristics

Among 782 patients contacted by email, 59 (7.5%) showed interest in participating in the study, and 48 (81%) of them responded to the survey. Among 448 patients contacted by phone calls, 61 (13.6%) showed interest, and 52 (85%) of them responded to the survey. Overall, the survey response rate was 8.1% (100/1230). There was no difference in age between patients who responded to the survey and patients who did not, including those who did not show interest in participating in the study (67.6 vs 67.7 years, *P*=.94). Of the patients contacted for this study and who did not want to participate, 73 provided reasons for nonparticipation. The common reasons included poor health condition (n=31, 42%), no interest (n=17, 23%), no time (n=11, 15%), and no access or uncomfortable with the use of technology (n=9, 12%).

Among 100 respondents, 45% (ie, 45 of the participants recruited by phone) completed the survey by phone and 55% completed it online. The respondents were on average 68 years old (SD 13 years), with 90% (90/100) White, 39% (39/100) women, and 88% (88/100) reporting having access to the internet or a mobile phone. As shown in Table 1, the rates of access to the internet (*P*<.001) and a smartphone (*P*<.001) were higher in online survey respondents than phone survey respondents. Among the 62 older participants (≥65 years old), 49 (79%) and 41 (66%) reported having access to the internet and a smartphone, respectively.

**Table 1:** Participant characteristics overall and by the survey delivery mode.

Characteristic		Total (N=100), n (%)	Survey delivery mode, n (%)		*P* value^a^
			Phone (n=45)	Online (n=55)	
**Age group**					.82
	<65 years	38 (38)	16 (36)	22 (40)	
	65-74 years	32 (32)	14 (31)	18 (33)	
	≥75 years	30 (30)	15 (33)	15 (27)	
**Gender**					.41
	Female	39 (39)	20 (44)	19 (35)	
	Male	59 (59)	24 (53)	35 (64)	
	Not reported	2 (2)	1 (2)	1 (2)	
**Race**					>.99
	White	90 (90)	39 (87)	51 (93)	
	Others	6 (6)	3 (7)	3 (5)	
	Not reported	4 (4)	3 (7)	1 (2)	
**Has access to the internet**					<.001^b^
	No	15 (15)	14 (31)	1 (2)	
	Yes	85 (85)	31 (69)	54 (98)	
**Has a smartphone**					<.001^b^
	No	25 (25)	19 (42)	6 (11)	
	Yes	75 (75)	26 (58)	49 (89)	

^a^Calculated by the Fisher exact test for categorical variables, using complete case analysis (ie, ignoring missing values for gender and race).

^b^Statistically significant (*P*<.05).

### Intention to Use the Symptom Monitoring App

All participants (N=100) responded to the intention-to-use survey item, with responses of strongly agree (n=19), agree (n=46), neutral (n=15), disagree (n=15), and strongly disagree (n=5). A total of 74 participants responded to the survey item “I plan to use this app as often as necessary,” with responses of strongly agree (n=22), agree (n=35), neutral (n=16), disagree (n=1), and strongly disagree (n=0). Among the 65 (65%) respondents with a positive intention (agree or strongly agree) to use the app, 53 (82%) agreed or strongly agreed that they would use the app as often as possible. Among the 73 respondents to the app design question, 28 (38%) preferred a mobile app, 30 (41%) preferred a web-based app, 14 (19%) liked both mobile and web-based apps, and 1 (1%) preferred another design (unspecified).

#### Intention to Use by Patient Characteristics

Among the 62 older participants (≥65 years old), 37 (60%) reported having the intention to use the app. As shown in Table 2, survey respondents aged 75 years or older had a lower rate of intention (ie, agree or strongly agree) to use the app (43%) than those in other age groups (74% for ages under 65 years and 75% for ages 65-74 years; Fisher exact test *P*=.02). There was no difference in the intention to use by gender or race. The rate of the intention to use the app was higher in respondents with access to the internet or a smartphone than those without access (72% vs 17%, *P*<.001).

The mean (Table 2) and median (Multimedia Appendix 3) scores of the intention to use and the distributions of the 5 levels of the intention to use (Multimedia Appendix 3), stratified by participant characteristics, showed similar patterns.

**Table 2:** Distribution of the intention to use a symptom monitoring app by patient characteristics and the survey delivery mode.

Variable^a^		Response score^b^, mean (SD)	Rate of a positive (agree or strongly agree) intention to use the app		
			n/N	%	*P* value^c^
All		3.6 (1.1)	65/100	65	
**Age group**					.02^d^
	<65 years	3.9 (0.8)	28/38	74	
	65-74 years	3.7 (1.1)	24/32	75	
	≥75 years	3.1 (1.3)	13/30	43	
**Gender**					>.99
	Female	3.6 (1.0)	25/39	64	
	Male	3.6 (1.2)	39/59	66	
**Race**					.66
	White	3.6 (1.1)	59/90	66	
	Others	3.3 (0.8)	3/6	50	
**Has access to technology (internet or a smartphone)**					<.001^d^
	No	2.2 (1.0)	2/12	17	
	Yes	3.8 (1.0)	63/88	72	
**Survey delivery mode**					.001^d^
	Phone	3.1 (1.3)	21/45	47	
	Online	4.0 (0.8)	44/55	80	

^a^The gender and race variables had 2 and 4 missing values, respectively.

^b^Scores assigned to the response options were as follows: 1, strongly disagree; 2, disagree; 3, neutral; 4, agree; 5, strongly agree.

^c^Calculated by the Fisher exact test for all the items.

^d^Statistically significant (*P*<.05).

#### Intention to Use by the Survey Delivery Mode

The rate of a positive intention to use the app (Table 2) was higher in online survey respondents than in phone survey respondents (80% vs 47%, *P*=.001). After adjusting for age and access to technology, the difference remained significant (adjusted odds ratio 3.07, 95% CI 1.20-7.88).

Similarly, the mean (Table 2) and median (Multimedia Appendix 3) scores of the intention to use were higher in online survey respondents (mean 4.0, median 4) than in phone survey respondents (mean 3.1, median 3).

### Facilitators and Barriers to Using the App

**Figure 1:**
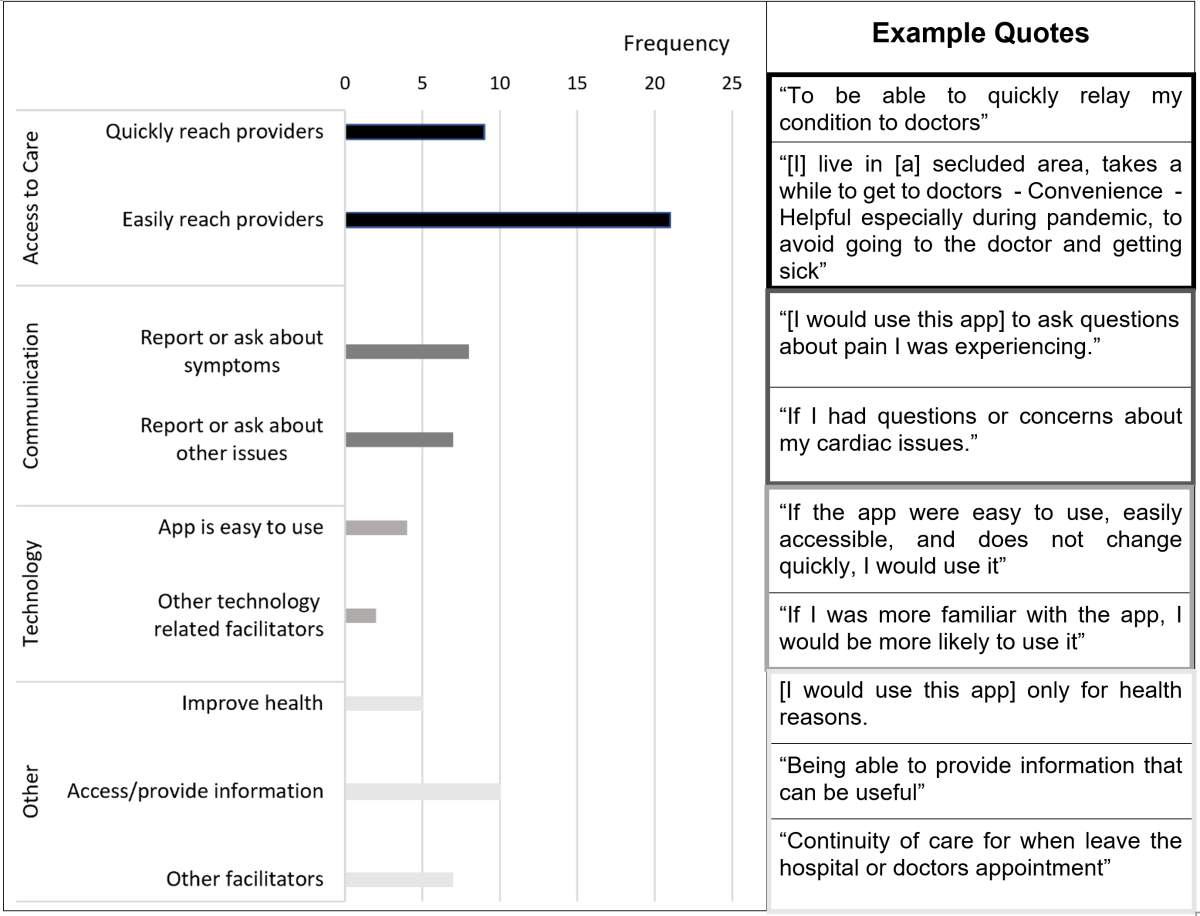
Facilitators to using a symptom monitoring app. Each segment was assigned a single code (ie, facilitator). We have provided an example quote for each code (in parallel to the bars in the figure). More example quotes are provided in Multimedia Appendix 2.

**Figure 2:**
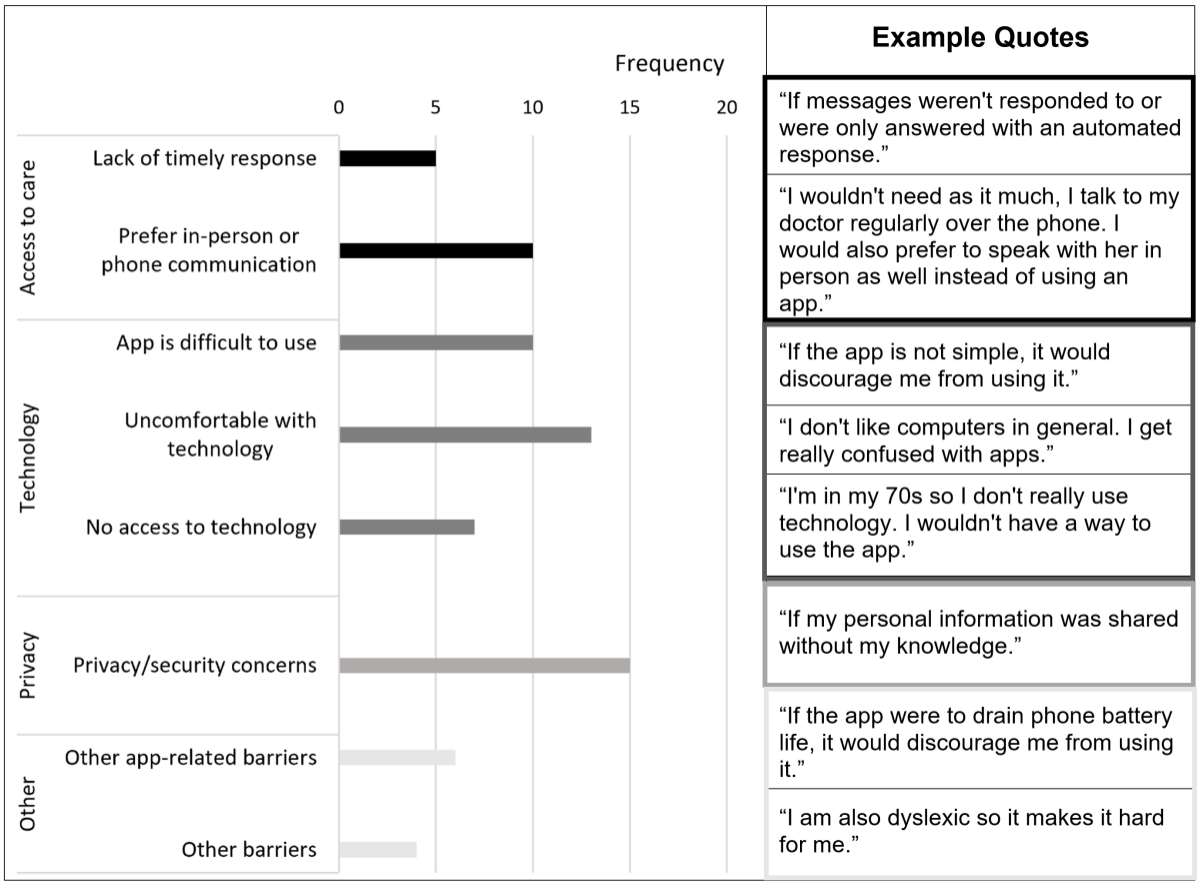
Barriers to using a symptom monitoring app. Each segment was assigned a single code (ie, barrier). We have provided an example quote for each code (in parallel to the bars in the figure). More example quotes are provided in Multimedia Appendix 2.

A total of 84 (84%) participants responded to the facilitator question, for which we identified 73 segments (from 66 participants) that described facilitators. A total of 80 (80%) participants responded to the barrier question, for which we identified 70 segments (from 63 participants) that described barriers. The analyses of these segments identified 9 facilitators or motivations (Figure 1) and 9 barriers (Figure 2). The major facilitators included (1) easily reaching providers, (2) accessing or providing information, (3) quickly reaching providers, and (4) consulting providers for symptoms. We distinguished between barriers 1 and 3, with barrier 1 focusing on convenience in care access (see code definition and more example quotes in Multimedia Appendix 2). The main barriers included (1) privacy/security concerns, (2) uncomfortable using technology, (3) user-unfriendly app interface, and (4) preference for in-person/phone care.

Among participants under 65 years, 87% (33/38) mentioned facilitators to app use, with the most noticeable one being “easily reach providers” (frequency of 14). Among participants aged 65 years or older, 53% (33/62) mentioned facilitators, with the most noticeable one being “access and provide information” (frequency of 8). Among participants under 65, 55% (21/38) mentioned barriers to app use, with the most noticeable one being “lack of timely response” (frequency of 5). Among participants aged 65 years or older, 65% (40/62) mentioned barriers, with the most noticeable one being “uncomfortable with technology” (frequency of 12).

## Discussion

### Principal Findings

This is the first study to assess the intention to use a postdischarge symptom monitoring app in ACS patients. We found that most (65/100, 65%) ACS patients had the intention to use an app to monitor and report postdischarge symptoms to providers. Compared with other participants, those aged 75 years or older or lacking access to technology (ie, internet and smartphones) had a lower intention to use the app. Furthermore, phone survey respondents had a lower intention to use the app than online survey respondents. Open-ended survey questions identified important facilitators (Figure 1) and barriers (Figure 2) to using the app in the following 4 domains: access to care, communication, technology, and privacy.

### Intention to Use Digital Symptom Monitoring in Older Patients With ACS

Although ACS patients are mostly older adults, we still found a high intention to use the symptom monitoring tool in this population. Specifically, 60% of older participants (≥65 years old) had the intention to use the app. Furthermore, the percentage of participants aged 65-74 years who had the intention to use the app (75%) was as high as that (74%) among younger participants. Our findings are compatible with previous findings on the intention to use health information technology, including symptom monitoring apps, in older adults [27,28,36-41]. For example, prior studies found that 46%-51% of participants older than 60 years would like to use a mobile app to track mental health conditions [27,28]. Other studies also found mobile symptom tracking apps acceptable for older patients with heart failure [38,39], and an app incorporating design features specific to older adults received high usability scores [39]. Similar to prior studies [27,42], we found that older participants had a lower intention to use the app, but we saw this pattern only in participants aged 75 years or older.

### Lack of an Email Address in the EHR: A Potential Indicator for a Low Intention to Use Digital Symptom Monitoring

For this study, we intentionally used phone calls to recruit patients who did not have an email address in the EHR. The absence of an email address may imply a lack of email access, infrequent use of email, or less comfort with sending and receiving emails. Most of these participants (ie, those without an email address in the EHR) chose to complete the survey over the phone and had a lower intention to use a symptom monitoring app, even after adjusting for age and access to technology. This suggests that a lack of an email address itself may be a useful predictor and provide meaningful information for health care teams making decisions about remote symptom monitoring postdischarge. In the future, this information (ie, lack of an email address in the EHR) can be used to purposefully sample key informants to help design and user test symptom monitoring apps and identify patients who may need greater training and support in app use.

### Patient-Perceived Facilitators and Barriers to Using Digital Symptom Monitoring

This study also identified important facilitators and barriers to using a symptom monitoring app in ACS patients. Prior studies found that perceived usefulness significantly influenced the intention to use medical apps in older patients [40,41,43,44]. Similarly, we found that the facilitators or motivations to using a symptom monitoring app mainly were related to perceived usefulness of the app, such as reaching health providers easily, accessing and providing health information, and consulting with providers regarding symptom management. The major barrier identified was patients’ concerns with privacy and security. This is common with digital health interventions and needs to be addressed from the perspectives of both the app and the users [45-48]. In addition to following the regulations and incorporating standard security features in app design [47,48], it is important to assess user opinions on desired privacy and security features in their local context [46,49]. In this study, we found that ACS patients were concerned about who will access their health information and the disclosure of their health information to a third party without their knowledge and authorization. Using hospital-authorized apps, clearly communicating with patients an app’s privacy statement, and providing options for choosing which information to disclose with whom may reduce this barrier. Similar to prior studies [25,26], we found that the most notable barrier for using the symptom app in older (≥65 years old) ACS patients is being uncomfortable using technology. Patient-centered app design, in-hospital training for app use, and app use support from caregivers may help reduce the barriers [50].

Previous studies found that patients sometimes have challenges in deciding when to use an app to report symptoms. For example, patients sometimes reported urgent issues via secure messaging services designed for communicating nonurgent issues [51-53]. In addition, prior studies found that ACS patients were more stressful about certain symptoms and 15% of patients developed stress disorder symptoms after ACS [54,55]. It is likely that some patients would unnecessarily seek acute care when experiencing nonurgent symptoms [56]. In this study, we did not find these issues to be a theme when analyzing patient-reported barriers to app use. However, it is important to communicate with patients about the appropriate use of a symptom monitoring app and how frequently providers would review or respond to patient reporting. Patient education on how to assess the severity of symptoms, for example, identifying typical ACS symptoms that need urgent care, is also relevant and may improve health care utilization.

### Implications on App Design and Development

Whether an intention to use a digital health app can translate into real use depends on many factors, such as app design and implementation strategies to support app use. In addition to general app design principles (eg, secure and easy to use), this study suggests additional considerations in app development for ACS patients. Specifically, we found that older age and lack of access to technology were associated with a low intention to use the app, and the most common barrier to app use in older adults was being uncomfortable using technology. This suggests that a multimodal strategy may be more effective in engaging these patients. For those who have nonsmart phones or are less comfortable using apps, text messaging may serve as an additional communication channel. Alternatively, app design may allow for the involvement of family members or caregivers in symptom tracking. In addition, accessible design principles for older adults may be incorporated by including a consistent and simple interface, making the most essential functionalities readily visible and available, and making it easier to “undo” an unintended action [57,58]. A co-creation approach that engages older patients in all stages of app development and user testing is also important for improving app adoption and user experience [59,60].

In this study, we also found that patients were motivated to use an app to easily reach providers. Therefore, the app should allow providers to easily access symptom reports, triage symptoms, and respond to patient symptoms and concerns. It is also critical to engage providers in all phases of app design and testing. App adoption will need to address how to integrate information from the app into the EHR, and assess the impact of the app on provider burden and clinical workflow [61,62].

### Limitations

Our sample was relatively small and from a health care system in 1 state, and most participants were non-Hispanic White. Therefore, our findings may not be generalizable to other settings. Constrained by the format of a survey study, participants’ responses to the open-ended survey questions were typically short and lacked detailed information about the contextual factors related to the perceived facilitators and barriers. We interpret these qualitative results based on the existing literature. In-depth qualitative studies are warranted to better understand certain barriers, such as the preference for in-person care and phone communication.

### Conclusions

We found a strong intention of using a symptom monitoring app postdischarge among ACS patients. However, this intent was lower in patients aged 75 years or older. Our survey identified barriers related to privacy and security, technology use, and the care delivery mode. Using hospital-authorized apps and in-hospital training may reduce the barriers. Further research is warranted to determine if such intent translates into app use, and better symptom management and health care quality.

## Appendix

Multimedia Appendix 1 Survey to assess the intention to use a symptom monitoring app.

Multimedia Appendix 2 Codebook and example quotes for facilitators and barriers to using a symptom monitoring app.

Multimedia Appendix 3 Intention to use a symptom monitoring app, stratified by patient characteristics and the survey delivery mode.

## Abbreviations

ACS: acute coronary syndrome
EHR: electronic health record

## References

[1] Virani SS, Alonso A, Benjamin EJ, Bittencourt MS, Callaway CW, Carson AP, Chamberlain AM, Chang AR, Cheng S, Delling FN, Djousse L, Elkind MSV, Ferguson JF, Fornage M, Khan SS, Kissela BM, Knutson KL, Kwan TW, Lackland DT, Lewis TT, Lichtman JH, Longenecker CT, Loop MS, Lutsey PL, Martin SS, Matsushita K, Moran AE, Mussolino ME, Perak AM, Rosamond WD, Roth GA, Sampson UKA, Satou GM, Schroeder EB, Shah SH, Shay CM, Spartano NL, Stokes A, Tirschwell DL, VanWagner LB, Tsao CW, American Heart Association Council on EpidemiologyPrevention Statistics CommitteeStroke Statistics Subcommittee (2020). Heart Disease and Stroke Statistics-2020 Update: A Report From the American Heart Association. Circulation.

[2] Dharmarajan K, Hsieh AF, Lin Z, Bueno H, Ross JS, Horwitz LI, Barreto-Filho JA, Kim N, Bernheim SM, Suter LG, Drye EE, Krumholz HM (2013). Diagnoses and timing of 30-day readmissions after hospitalization for heart failure, acute myocardial infarction, or pneumonia. JAMA.

[3] Wasfy JH, Strom JB, O'Brien C, Zai AH, Luttrell J, Kennedy KF, Spertus JA, Zelevinsky K, Normand ST, Mauri L, Yeh RW (2014). Causes of short-term readmission after percutaneous coronary intervention. Circ Cardiovasc Interv.

[4] Southern DA, Ngo J, Martin B, Galbraith PD, Knudtson ML, Ghali WA, James MT, Wilton SB (2014). Characterizing types of readmission after acute coronary syndrome hospitalization: implications for quality reporting. J Am Heart Assoc.

[5] Kwok CS, Shah B, Al-Suwaidi J, Fischman DL, Holmvang L, Alraies C, Bagur R, Nagaraja V, Rashid M, Mohamed M, Martin GP, Kontopantelis E, Kinnaird T, Mamas M (2019). Timing and Causes of Unplanned Readmissions After Percutaneous Coronary Intervention: Insights From the Nationwide Readmission Database. JACC Cardiovasc Interv.

[6] Shah M, Patil S, Patel B, Agarwal M, Davila CD, Garg L, Agrawal S, Kapur NK, Jorde UP (2018). Causes and Predictors of 30-Day Readmission in Patients With Acute Myocardial Infarction and Cardiogenic Shock. Circ Heart Fail.

[7] Iribarne A, Chang H, Alexander JH, Gillinov AM, Moquete E, Puskas JD, Bagiella E, Acker MA, Mayer ML, Ferguson TB, Burks S, Perrault LP, Welsh S, Johnston KC, Murphy M, DeRose JJ, Neill A, Dobrev E, Baio KT, Taddei-Peters W, Moskowitz AJ, O'Gara PT (2014). Readmissions after cardiac surgery: experience of the National Institutes of Health/Canadian Institutes of Health research cardiothoracic surgical trials network. Ann Thorac Surg.

[8] Enderlin CA, McLeskey N, Rooker JL, Steinhauser C, D'Avolio D, Gusewelle R, Ennen KA (2013). Review of current conceptual models and frameworks to guide transitions of care in older adults. Geriatr Nurs.

[9] (2019). Medicare Program; CY 2020 Revisions to Payment Policies Under the Physician Fee Schedule and Other Changes to Part B Payment Policies; Medicare Shared Savings Program Requirements; Medicaid Promoting Interoperability Program Requirements for Eligible Professionals; Establishment of an Ambulance Data Collection System; Updates to the Quality Payment Program; Medicare Enrollment of Opioid Treatment Programs and Enhancements to Provider Enrollment Regulations Concerning Improper Prescribing and Patient Harm; and Amendments to Physician Self-Referral Law Advisory Opinion Regulations Final Rule; and Coding and Payment for Evaluation and Management, Observation and Provision of Self-Administered Esketamine Interim Final Rule. Centers for Medicare & Medicaid Services.

[10] Jack B, Paasche-Orlow M, Mitchell S, Forsythe S, Martin J, Brach C (2012). An overview of the Re-Engineered Discharge (RED) Toolkit (Prepared by Boston University under Contract No. HHSA290200600012i). Boston University.

[11] Coleman EA, Parry C, Chalmers S, Min S (2006). The care transitions intervention: results of a randomized controlled trial. Arch Intern Med.

[12] Hirschman KB, Shaid E, McCauley K, Pauly MV, Naylor MD (2015). Continuity of Care: The Transitional Care Model. Online J Issues Nurs.

[13] Burke RE, Guo R, Prochazka AV, Misky GJ (2014). Identifying keys to success in reducing readmissions using the ideal transitions in care framework. BMC Health Serv Res.

[14] Bloink J, Adler KG (2013). Transitional care management services: new codes, new requirements. Fam Pract Manag.

[15] Felix HC, Seaberg B, Bursac Z, Thostenson J, Stewart MK (2015). Why do patients keep coming back? Results of a readmitted patient survey. Soc Work Health Care.

[16] Bennett AV, Jensen RE, Basch E (2012). Electronic patient-reported outcome systems in oncology clinical practice. CA Cancer J Clin.

[17] Jensen RE, Snyder CF, Abernethy AP, Basch E, Potosky AL, Roberts AC, Loeffler DR, Reeve BB (2014). Review of electronic patient-reported outcomes systems used in cancer clinical care. J Oncol Pract.

[18] Masterson Creber RM, Maurer MS, Reading M, Hiraldo G, Hickey KT, Iribarren S (2016). Review and Analysis of Existing Mobile Phone Apps to Support Heart Failure Symptom Monitoring and Self-Care Management Using the Mobile Application Rating Scale (MARS). JMIR Mhealth Uhealth.

[19] Wang K, Varma DS, Prosperi M (2018). A systematic review of the effectiveness of mobile apps for monitoring and management of mental health symptoms or disorders. J Psychiatr Res.

[20] Menni C, Valdes AM, Freidin MB, Sudre CH, Nguyen LH, Drew DA, Ganesh S, Varsavsky T, Cardoso MJ, El-Sayed Moustafa JS, Visconti A, Hysi P, Bowyer RCE, Mangino M, Falchi M, Wolf J, Ourselin S, Chan AT, Steves CJ, Spector TD (2020). Real-time tracking of self-reported symptoms to predict potential COVID-19. Nat Med.

[21] Basch E, Deal AM, Kris MG, Scher HI, Hudis CA, Sabbatini P, Rogak L, Bennett AV, Dueck AC, Atkinson TM, Chou JF, Dulko D, Sit L, Barz A, Novotny P, Fruscione M, Sloan JA, Schrag D (2016). Symptom Monitoring With Patient-Reported Outcomes During Routine Cancer Treatment: A Randomized Controlled Trial. J Clin Oncol.

[22] Denis F, Basch E, Septans A, Bennouna J, Urban T, Dueck AC, Letellier C (2019). Two-Year Survival Comparing Web-Based Symptom Monitoring vs Routine Surveillance Following Treatment for Lung Cancer. JAMA.

[23] Basch E, Deal AM, Dueck AC, Scher HI, Kris MG, Hudis C, Schrag D (2017). Overall Survival Results of a Trial Assessing Patient-Reported Outcomes for Symptom Monitoring During Routine Cancer Treatment. JAMA.

[24] Wolf A, Fors A, Ulin K, Thorn J, Swedberg K, Ekman I (2016). An eHealth Diary and Symptom-Tracking Tool Combined With Person-Centered Care for Improving Self-Efficacy After a Diagnosis of Acute Coronary Syndrome: A Substudy of a Randomized Controlled Trial. J Med Internet Res.

[25] Gitlow L (2014). Technology Use by Older Adults and Barriers to Using Technology. Physical & Occupational Therapy In Geriatrics.

[26] Vaportzis E, Clausen MG, Gow AJ (2017). Older Adults Perceptions of Technology and Barriers to Interacting with Tablet Computers: A Focus Group Study. Front Psychol.

[27] Torous J, Friedman R, Keshavan M (2014). Smartphone ownership and interest in mobile applications to monitor symptoms of mental health conditions. JMIR Mhealth Uhealth.

[28] Torous J, Chan SR, Yee-Marie Tan S, Behrens J, Mathew I, Conrad EJ, Hinton L, Yellowlees P, Keshavan M (2014). Patient Smartphone Ownership and Interest in Mobile Apps to Monitor Symptoms of Mental Health Conditions: A Survey in Four Geographically Distinct Psychiatric Clinics. JMIR Ment Health.

[29] van Velsen L, van der Geest T, van de Wijngaert L, van den Berg S, Steehouder M (2015). Personalization has a Price, Controllability is the Currency: Predictors for the Intention to use Personalized eGovernment Websites. Journal of Organizational Computing and Electronic Commerce.

[30] Jansen-Kosterink S, Hurmuz M, den Ouden M, van Velsen L (2020). Predictors to use mobile apps for monitoring COVID-19 symptoms and contact tracing: A survey among Dutch citizens. medRxiv.

[31] Harris PA, Taylor R, Minor BL, Elliott V, Fernandez M, O'Neal L, McLeod L, Delacqua G, Delacqua F, Kirby J, Duda SN, REDCap Consortium (2019). The REDCap consortium: Building an international community of software platform partners. J Biomed Inform.

[32] Harris PA, Taylor R, Thielke R, Payne J, Gonzalez N, Conde JG (2009). Research electronic data capture (REDCap)--a metadata-driven methodology and workflow process for providing translational research informatics support. J Biomed Inform.

[33] Hsieh H, Shannon SE (2005). Three approaches to qualitative content analysis. Qual Health Res.

[34] Vaismoradi M, Turunen H, Bondas T (2013). Content analysis and thematic analysis: Implications for conducting a qualitative descriptive study. Nurs Health Sci.

[35] Elo S, Kyngäs H (2008). The qualitative content analysis process. J Adv Nurs.

[36] Parker SJ, Jessel S, Richardson JE, Reid MC (2013). Older adults are mobile too!Identifying the barriers and facilitators to older adults' use of mHealth for pain management. BMC Geriatr.

[37] Hung L, Lyons JG, Wu C (2020). Health information technology use among older adults in the United States, 2009-2018. Curr Med Res Opin.

[38] Portz JD, Vehovec A, Dolansky MA, Levin JB, Bull S, Boxer R (2018). The Development and Acceptability of a Mobile Application for Tracking Symptoms of Heart Failure Among Older Adults. Telemed J E Health.

[39] Reading Turchioe M, Grossman LV, Baik D, Lee CS, Maurer MS, Goyal P, Safford MM, Masterson Creber RM (2020). Older Adults Can Successfully Monitor Symptoms Using an Inclusively Designed Mobile Application. J Am Geriatr Soc.

[40] Askari M, Klaver NS, van Gestel TJ, van de Klundert J (2020). Intention to use Medical Apps Among Older Adults in the Netherlands: Cross-Sectional Study. J Med Internet Res.

[41] de Veer AJE, Peeters JM, Brabers AEM, Schellevis FG, Rademakers JJDJM, Francke AL (2015). Determinants of the intention to use e-Health by community dwelling older people. BMC Health Serv Res.

[42] Sohn A, Speier W, Lan E, Aoki K, Fonarow G, Ong M, Arnold C (2019). Assessment of Heart Failure Patients' Interest in Mobile Health Apps for Self-Care: Survey Study. JMIR Cardio.

[43] Cajita MI, Hodgson NA, Budhathoki C, Han H (2017). Intention to Use mHealth in Older Adults With Heart Failure. J Cardiovasc Nurs.

[44] Zhang Y, Liu C, Luo S, Xie Y, Liu F, Li X, Zhou Z (2019). Factors Influencing Patients' Intentions to Use Diabetes Management Apps Based on an Extended Unified Theory of Acceptance and Use of Technology Model: Web-Based Survey. J Med Internet Res.

[45] Dennison L, Morrison L, Conway G, Yardley L (2013). Opportunities and challenges for smartphone applications in supporting health behavior change: qualitative study. J Med Internet Res.

[46] Zhou L, Bao J, Watzlaf V, Parmanto B (2019). Barriers to and Facilitators of the Use of Mobile Health Apps From a Security Perspective: Mixed-Methods Study. JMIR Mhealth Uhealth.

[47] Arora S, Yttri J, Nilse W (2014). Privacy and Security in Mobile Health (mHealth) Research. Alcohol Res.

[48] Morera EP, de la Torre Díez I, Garcia-Zapirain B, López-Coronado M, Arambarri J (2016). Security Recommendations for mHealth Apps: Elaboration of a Developer's Guide. J Med Syst.

[49] Atienza AA, Zarcadoolas C, Vaughon W, Hughes P, Patel V, Chou WS, Pritts J (2015). Consumer Attitudes and Perceptions on mHealth Privacy and Security: Findings From a Mixed-Methods Study. J Health Commun.

[50] Veinot TC, Mitchell H, Ancker JS (2018). Good intentions are not enough: how informatics interventions can worsen inequality. J Am Med Inform Assoc.

[51] North F, Crane SJ, Stroebel RJ, Cha SS, Edell ES, Tulledge-Scheitel SM (2013). Patient-generated secure messages and eVisits on a patient portal: are patients at risk?. J Am Med Inform Assoc.

[52] Lanham HJ, Leykum LK, Pugh JA (2018). Examining the Complexity of Patient-Outpatient Care Team Secure Message Communication: Qualitative Analysis. J Med Internet Res.

[53] Shimada SL, Petrakis BA, Rothendler JA, Zirkle M, Zhao S, Feng H, Fix GM, Ozkaynak M, Martin T, Johnson SA, Tulu B, Gordon HS, Simon SR, Woods SS (2017). An analysis of patient-provider secure messaging at two Veterans Health Administration medical centers: message content and resolution through secure messaging. J Am Med Inform Assoc.

[54] Ayers S, Copland C, Dunmore E (2009). A preliminary study of negative appraisals and dysfunctional coping associated with post-traumatic stress disorder symptoms following myocardial infarction. Br J Health Psychol.

[55] Edmondson D, Rieckmann N, Shaffer JA, Schwartz JE, Burg MM, Davidson KW, Clemow L, Shimbo D, Kronish IM (2011). Posttraumatic stress due to an acute coronary syndrome increases risk of 42-month major adverse cardiac events and all-cause mortality. J Psychiatr Res.

[56] Kwok CS, Wong CW, Shufflebotham H, Brindley L, Fatima T, Shufflebotham A, Barker D, Pawala A, Heatlie G, Mamas MA (2017). Early Readmissions After Acute Myocardial Infarction. Am J Cardiol.

[57] Fisk A, Czaja S, Rogers W, Charness N, Sharit J (2019). Designing for Older Adults: Principles and Creative Human Factors Approaches.

[58] Farage MA, Miller KW, Ajayi F, Hutchins D (2012). Design principles to accommodate older adults. Glob J Health Sci.

[59] Mansson L, Wiklund M, Öhberg F, Danielsson K, Sandlund M (2020). Co-Creation with Older Adults to Improve User-Experience of a Smartphone Self-Test Application to Assess Balance Function. Int J Environ Res Public Health.

[60] Mannheim I, Schwartz E, Xi W, Buttigieg SC, McDonnell-Naughton M, Wouters EJM, van Zaalen Y (2019). Inclusion of Older Adults in the Research and Design of Digital Technology. Int J Environ Res Public Health.

[61] Mishuris RG, Yoder J, Wilson D, Mann D (2016). Integrating data from an online diabetes prevention program into an electronic health record and clinical workflow, a design phase usability study. BMC Med Inform Decis Mak.

[62] Borycki E, Kushniruk A, Nohr C, Takeda H, Kuwata S, Carvalho C, Bainbridge M, Kannry J (2013). Usability Methods for Ensuring Health Information Technology Safety: Evidence-Based Approaches. Contribution of the IMIA Working Group Health Informatics for Patient Safety. Yearb Med Inform.

