# Supplementary material for: Understanding Patients’ Intention to Use Digital Health Apps That Support Postdischarge Symptom Monitoring by Providers Among Patients With Acute Coronary Syndrome: Survey Study
Source: JMIR Hum Factors. 2022 Mar 7;9(1):e34452. doi: 10.2196/34452 (PMC8938838; doi:10.2196/34452)
Supplement: Multimedia Appendix 1 [file humanfactors_v9i1e34452_app1.docx]

## Multimedia Appendix 1. Survey to assess the intention to use a symptom monitoring app*.*

Thank you for participating in this short survey.

In collaboration with UMMHC cardiology clinics, our study team plans to develop an app to help patients to communicate with healthcare providers after they leave the hospital. The app is a software tool that can be used on mobile phones or computers. It will allow patients to report their symptoms (if any) to their health providers weekly or when they want to tell their healthcare providers about their symptoms.

[online survey] Below you will find our questions about your thoughts or expectations about the app and some basic information about you. For each statement, please check the answer that best matches your thoughts or expectations. Your answers will be helpful for our app design. Thank you!

[phone survey: I am going to ask you questions about your thoughts or expectations about the app and some basic information about you. For each statement, please select the answer that best matches your thoughts or expectations. Your answers will be helpful for our app design. Thank you!]

1. I have a smartphone:

o Yes

o No

2. I can access the internet (through my mobile phone or computer) at home:

o Yes

o No

3. If this app were available to me, I would use it:

o Strongly agree

o Agree

o Neutral

o Disagree

o Strongly disagree

If you are interested in using the app in future when it is available, please answer questions #4 and #5. If not, please skip to question #6.

4. I plan to use this app as often as necessary:

o Strongly agree

o Agree

o Neutral

o Disagree

o Strongly disagree

5. I’d like the app to be designed as:

o A mobile app (a software application that can be used on smartphones and tablets)

o A web-based app (a software application that can be accessed from the web browser on computers, smartphones and tablets)

o Other

We would like to know a little more about your thoughts on using this app.

6. What is the main reason(s) that would motivate you to use this app? (optional)

7. What is the main reason(s) that would discourage you from using this app? (optional)

We now would like to know a little more about you.

8. What is your gender?

o Female

o Male

o Non-binary

o Prefer not to say

9. What is your age?

o Below 25

o 25-44

o 45-64

o 65 and above

o Prefer not to say

10. What is your race? (please select all that apply)

o American Indian or Alaska Native

o Asian

o Black or African American

o Hispanic/Latinx

o Native Hawaiian or Other Pacific Islander

o White

o Prefer not to say
