# Supplementary material for: Understanding Patients’ Intention to Use Digital Health Apps That Support Postdischarge Symptom Monitoring by Providers Among Patients With Acute Coronary Syndrome: Survey Study
Source: JMIR Hum Factors. 2022 Mar 7;9(1):e34452. doi: 10.2196/34452 (PMC8938838; doi:10.2196/34452)
Supplement: Multimedia Appendix 2 [file humanfactors_v9i1e34452_app2.docx]

## Multimedia Appendix 2. Codebook and example quotes for facilitators and barriers to using a symptom monitoring app*.*

**Table S1. Codebook and example quotes for facilitators to using a symptom monitoring app.**

| **Code/Category (frequency)** | **Definition** | **Example Quotes** |
| --- | --- | --- |
| 1. Access to care | Patients’ access to care |  |
| 1.1 Quickly reach providers (9) | Can reach health care providers in a timely manner to report symptoms/issues or seek care/help | “Timely and responsive support for my health [would motivate me to use the app.]”  “To be able to quickly relay my condition to doctors”  “Faster less stressful communications with my doctors” |
| 1.2 Easily reach providers (21) | Can reach health care providers easily or conveniently to report symptoms/issues or seek care/help | “The main motivation for me would be to have the ability to contact my provider from my phone if necessary when traveling and unable to get to my computer.”  “It would help me communicate as I've been home more often due to the pandemic and my heart attack two year ago. It would also help me to avoid hospitals frequently.”  “[I] live in [a] secluded area, takes a while to get to doctors - Convenience - Helpful especially during pandemic, to avoid going to the doctor and getting sick” |
| 2. Communication | Patient-provider communication of symptoms or other issues |  |
| 2.1 Report/ask about  symptoms (8) | Communicate/report symptoms or ask questions specific to symptoms | “An application would be a more efficient system to report symptoms and allow health care providers to respond.”  “If I have symptoms after discharge and wanted to communicate with doctor.”  “[I would use this app] to ask questions about pain I was experiencing.” |
| 2.2 Report/ask about other  issues (7) | Communicate/report issues or ask questions nonspecific to symptoms | “If I had questions or concerns about my cardiac issues.”  “Casual questions/asking about medications.” |
| 3. Technology | Technology-related factors |  |
| 3.1 App is easy to use (4) | The app is easy to use | “If the app were easy to use, easily accessible, and does not change quickly, I would use it”  “If it was easy to use”  “Because it sounds easy/easy to use” |
| 3.2 Other technology-related  factors (2) | Other technology-related factors, such as having access to the internet or the app, having skills to use the app, etc. | “If I was more familiar with the app, I would be more likely to use it” |
| 4. Other | Factors not covered by categories 1-3 |  |
| 4.1 Improve Health (5) | Patient wants to use the app to improve their health in general | “[I would use this app] only for health reasons.”  “Medical purposes” |
| 4.2 Access or provide  information (10) | Can view information from health providers or provide information/feedback to health providers | “I would use it to check updates.”  “Easier access to information”  “Being able to provide information that can be useful” |
| 4.3 Other facilitators (7) | Facilitators not covered by other codes | “Continuity of care for when leave the hospital or doctors appointment.”  “Curiosity” |

Note: Each segment was assigned a single code or subcategory (i.e., barrier or facilitator). Some responses contained multiple segments and thus multiple facilitators (or barriers). We provided frequency and example quotes for each code.

**Table S2. Codebook and example quotes for barriers to using a symptom monitoring app.**

| **Code/Category (frequency)** | **Definition** | **Example Quotes** |
| --- | --- | --- |
| 1. Technology | Technology-related factors |  |
| 1.1 App is difficult to use (10) | The app is not user friendly or easy to use | “If the app is not simple, it would discourage me from using it.”  “An app that is not well laid out would discourage my use.  “If it is not user friendly” |
| 1.2 Uncomfortable with  technology (13) | Patients lack skills or feel uncomfortable to use technology (computer, smartphone, tablet) | “I don't like computers in general. I get really confused with apps.”  “Not using technology often makes me feel uncomfortable when I do have to use it.”  “Able to text and read text messages, but this would fall outside/beyond that scope.” |
| 1.3 No access to technology  or internet (7) | No access to technology (computer, smartphone, tablet, internet) that would allow them to be able to use the app | “I'm in my 70s so I don't really use technology. I wouldn't have a way to use the app.”  “I don’t have a computer at home”  “I don't use the Internet, I never really had an interest for it.” |
| 2. Privacy/security | Factors related to privacy/security |  |
| 2.1 Privacy/security concerns (15) | Concerns of breaches and leaking personal information or disclosure of personal information to third parties (e.g., insurance company) | “My concerns is maintaining patient confidentiality along with good security.”  “If my personal information was shared without my knowledge.”  “Too many people accessing my info.” |
| 3. Access to care | Factors related to access to care |  |
| 3.1 Lack of timely response (5) | Lacking response or having delayed response from health providers to patient’s inquiry | “Lack of response from doctor/provider.”  “Length of time for response from physician/physicians office.”  “If messages weren't responded to or were only answered with an automated response.” |
| 3.2 Prefer in-person/phone  communication (10) | Preferring face-to-face or phone communication with health providers about health-related issues | “I wouldn't need as it much, I talk to my doctor regularly over the phone. I would also prefer to speak with her in person as well instead of using an app.”  “I just want to see/talk with my doctor.”  “If serious, wouldn't use app, but would contact Dr. by phone.” |
| 4. Other | Factors not covered by categories 1-3 |  |
| 4.1 Other app-related barriers (6) | Barriers related to app, such as app fee, having too many apps on phone, etc. | “Would prefer not to have additional apps on my phone”  “If the app were to drain phone battery life, it would discourage me from using it.”  “Charging a fee” |
| 4.2 Other barriers (4) | Barriers not covered by other codes | “I am also dyslexic so it makes it hard for me.”  “Get into too much trouble.” |

Note: Each segment was assigned a single code or subcategory (i.e., barrier or facilitator). Some responses contained multiple segments and thus multiple facilitators (or barriers). We provided frequency and example quotes for each code.
