# Supplementary material for: Understanding Patients’ Intention to Use Digital Health Apps That Support Postdischarge Symptom Monitoring by Providers Among Patients With Acute Coronary Syndrome: Survey Study
Source: JMIR Hum Factors. 2022 Mar 7;9(1):e34452. doi: 10.2196/34452 (PMC8938838; doi:10.2196/34452)
Supplement: Multimedia Appendix 3 [file humanfactors_v9i1e34452_app3.docx]

## Multimedia Appendix 3. Intention to use a symptom monitoring app, stratified by patient characteristics and the survey delivery mode*.*

|  | Intention to Use a Symptom Monitoring App | | | | | | |
| --- | --- | --- | --- | --- | --- | --- | --- |
|  | Median [Interquartile range]^a^ | Strongly agree | Agree | Neutral | Disagree | Strongly disagree | *P* value^b^ |
|  |  | n =19 | n = 46 | n =15 | n =15 | n = 5 |  |
|  |  | n (%) | n (%) | n (%) | n (%) | n (%) |  |
| **Age group** |  |  |  |  |  |  | .02* |
| < 65 years | 4 [3,5] | 10 (53) | 18 (39) | 8 (53) | 2 (13) | 0 (0) |  |
| 65-74 years | 4 [3.5,4] | 5 (26) | 19 (41) | 2 (13) | 4 (27) | 2 (40) |  |
| ≥ 75 years | 3 [2,4] | 4 (21) | 9 (20) | 5 (33) | 9 (60) | 3 (60) |  |
| **Gender** |  |  |  |  |  |  | .91 |
| Female | 4 [3,4] | 7 (37) | 18 (40) | 7 (47) | 6 (43) | 1 (20) |  |
| Male | 4 [3,4] | 12 (63) | 27 (60) | 8 (53) | 8 (57) | 4 (80) |  |
| **Race** |  |  |  |  |  |  | .60 |
| White | 4 [3,4] | 19 (100) | 40 (93) | 13 (87) | 13 (93) | 5 (100) |  |
| Other | 3.5 [3,4] | 0 (0) | 3 (7) | 2 (13) | 1 (7) | 0 (0) |  |
| **Has access to technology (internet or smartphone)** |  |  |  |  |  |  | <.001* |
| No | 2 [1.5,2.5] | 0 (0) | 2 (4) | 1 (7) | 6 (40) | 3 (60) |  |
| Yes | 4 [3,4] | 19 (100) | 44 (96) | 14 (93) | 9 (60) | 2 (40) |  |
| **Survey delivery mode** |  |  |  |  |  |  | <.001* |
| phone | 3 [2,4] | 5 (26) | 16 (35) | 6 (40) | 13 (87) | 5 (100) |  |
| online | 4 [4,5] | 14 (74) | 30 (65) | 9 (60) | 2 (13) | 0 (0) |  |

^a^ numeric values assigned to the response options: 1 – strongly disagree, 2 – disagree, 3 – neutral, 4 – agree, 5 – strongly agree.

^b^ *P*-values were calculated by Fisher’s exact test.

* indicates statistically significant (*P*<.05).
